# Flexible Thermoelectric Device Based on Protrusion-Structured Liquid Metal Elastomer for Gravity Heat Pipe

**DOI:** 10.3390/mi15050592

**Published:** 2024-04-29

**Authors:** Xiaogang Zhang, Xinghua Zhang, Shaocheng Ge, Bailin Zhang, Dongguang Zhang, Jiayi Yang

**Affiliations:** 1 College of Safety and Emergency Management Engineering, Taiyuan University of Technology, Jinzhong 030600, China; 2 College of Mechanical and Vehicle Engineering, Taiyuan University of Technology, Taiyuan 030024, China; 3 College of Computer Science and Technology, Xi’an University of Science and Technology, Xi’an 710054, China

**Keywords:** soft devices, temperature monitoring, thermal conductivity, flexible electronics

## Abstract

Monitoring the temperature of the coal gangue mountains is fundamental to preventing their spontaneous combustion. However, the existing temperature monitoring systems fail to achieve stable, pollution-free temperature monitoring without affecting vegetation growth in these mountains. To address this issue, this work proposes a flexible thermoelectric device (FTD) based on a protrusion-structured liquid metal elastomer (LME). Utilizing a high-thermal-conductivity LME, the FTD adheres closely to the surface of the gravity heat pipe (GHP), ensuring compatibility between FTD and the curved surface of the GHP. Simultaneously, employing a low-thermal-conductivity elastomer helps concentrate heat onto FTD, thereby enhancing thermoelectric power generation efficiency. Additionally, the impact of the shape, size, and height of the protrusion structure at the cold end of the GHP on its efficiency was also investigated. The practical application of FTD on GHP was demonstrated.

## 1. Introduction

Coal gangue, a hard, low-carbon, blackish-gray rock occurring alongside coal seams during the coal formation process, represents a substantial solid waste product—constituting approximately 15% to 20% of the total coal output [1]. Despite its abundance, coal gangue lacks significant economic value and is commonly accumulated to form coal gangue mountains, primarily for land conservation purposes by mining enterprises. However, these accumulated coal gangue masses are susceptible to spontaneous combustion due to internal oxidation processes [2]. This combustion leads to the release of harmful gases, such as SO_2_, H_2_S, CO, and particulate matter, posing significant ecological threats to the surrounding areas and raising concerns about safety and human health within mining regions [3].

Monitoring ground temperatures in coal gangue mountains is paramount to preventing spontaneous combustion [1]. Typically, this monitoring is achieved through hardware circuits and thermocouples. However, existing monitoring systems often rely on dry cell batteries for power, resulting in short operational lifespans, frequent replacements, and environmental pollution due to battery disposal [4]. Apart from dry cell battery power supply, solar-powered systems, while an alternative, suffer from dependency on adequate external lighting, weather influence, and large panel sizes potentially impeding vegetation growth [5]. Therefore, there is an urgent need to explore a new power supply method that can ensure high stability and pollution-free ground temperature monitoring without affecting the vegetation growth on coal gangue mountains [6].

Gravity heat pipes (GHPs), comprising sealed metal pipes with a working fluid, offer a means to transfer heat from coal gangue mountains’ surfaces to lower temperatures within [7,8]. The high temperatures, exceeding 100 °C, on GHP surfaces present an opportunity to harness thermoelectric modules to convert this heat into electricity for powering ground temperature monitoring systems without impacting local vegetation [9,10,11]. However, conventional rigid thermoelectric modules struggle to adapt to GHPs, limiting their effectiveness [12,13,14].

Liquid metal (LM) refers to metals that are in a liquid state at room temperature. Among these, gallium-based LMs have attracted widespread attention due to their almost zero vapor pressure, fluidity, high electrical conductivity, high thermal conductivity, and non-toxicity. Two commonly used examples include eutectic gallium-indium (EGaIn), which is composed of 75% gallium and 25% indium by weight and has a melting point of 15.7 °C, and Galinstan, a eutectic alloy typically made up of 68% gallium, 22% indium, and 10% tin by weight, with a melting point of approximately −19 °C. Mixing LMs with soft silicone by shearing force can significantly enhance its thermal conductivity without sacrificing the inherent flexibility of the silicone, making it an ideal material for flexible thermal conductors [15,16].

**Figure 1:**
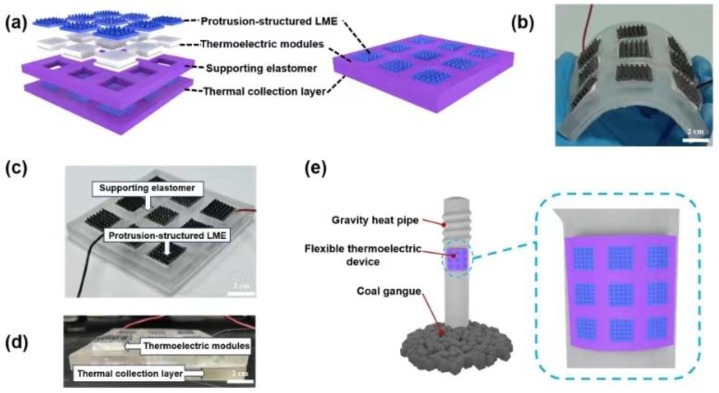
Schematic diagram and photos of a flexible thermoelectric device. (**a**) Exploded view of FTD structure; (**b**–**d**) physical photos of FTD; (**e**) schematic diagram of FTD application on gravity heat pipe.

This work proposes a flexible thermoelectric device (FTD) based on a protrusion-structured liquid metal elastomer (LME) for GHP applications. The FTD comprises a protrusion-structured LME, thermoelectric modules, a supporting elastomer, and a thermal collection layer, as shown in Figure 1a. The FTD has the advantages of high flexibility and efficiency. This work investigates the relationship between the volume fraction of LM in the elastomer and thermal conductivity, examines the effect of the protrusion-structured LME’s morphology on the temperature difference and open-circuit voltage of the thermoelectric modules. Finally, the FTD was attached to the surface of a GHP, which powered a ground temperature measurement system, demonstrating the potential application of the FTD.

## 2. Results and Discussion

The FTD comprises a protrusion-structured LME, thermoelectric modules, a supporting elastomer, and a thermal collection layer, as illustrated in Figure 1a. Benefiting from the high thermal conductivity of the LME, the protrusion-structured LME increases the temperature difference between the hot and cold sides of the thermoelectric modules and thus improves power generation efficiency. The interface where the thermoelectric modules are in contact with the air utilizes a flexible protrusion-structured LME for encapsulation, which not only possesses high thermal conductivity but also promotes heat dissipation in the air [17]. The thermoelectric modules are commercially available and can be adapted to the shape of curved objects. The supporting elastomer is made of PDMS and wraps around the thermoelectric module, serving as the support structure for the FTD. In addition, the support elastomer concentrates heat transmission through the thermoelectric modules, thereby enhancing power generation efficiency [18]. The thermal collection layer is composed of pure PDMS and LME, which encloses the contact area between the thermoelectric modules and the GHP using LME. The LME part of the thermal collection layer is in contact with the thermoelectric module, while the remaining part contacts the supporting elastomer. This configuration can direct the heat flow towards the thermoelectric module for efficient thermal energy conversion. The FTD exhibits flexibility and conformality to curved surfaces, as shown in Figure 1b. An optical photograph of the FTD is shown in Figure 1c, and the device contains nine thermoelectric modules connected in series. The dimensions of the FTD device are 15 cm × 15 cm. Each thermoelectric module is 2 cm × 2 cm. The LME appears black, while the pure elastomer is transparent white. The LM we used in this work is Galinstan, which consists of 68.5% gallium, 21.5% indium, and 10% tin by weight. It has a melting point of −19 °C.

**Figure 2:**
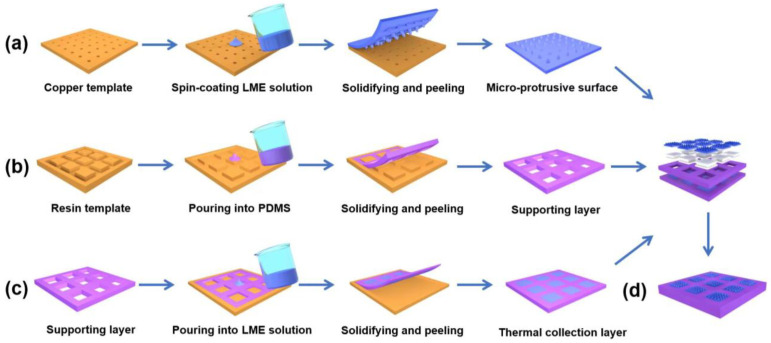
Fabrication process of FTD. (**a**) Schematic diagram of the fabrication process for protrusion-structured LME; (**b**) schematic diagram of the fabrication process for the support layer; (**c**) schematic diagram of the fabrication process for the heat collection layer; (**d**) schematic diagram of the FTD.

The fabrication process of the FTD based on the protrusion-structured LME is illustrated in Figure 2. We used polydimethylsiloxane (PDMS) as the elastomer for constructing the FTD. The PDMS solution is mixed in a 10:1 ratio of base and curing agent. Then, the PDMS solution was poured into the 3D-printed epoxy model. After curing in an oven at 80 °C for 4 h, we obtained the PDMS with the desired structure. As for the fabrication process of LME, before pouring PDMS into the mold, different volume fractions (*ϕ*) of LM are added to the as-prepared PDMS solution. This mixture is uniformly stirred using a shear mixer to obtain a homogeneous LME solution. After pouring the LEM solution into the molds, LMEs with designed structures were fabricated by curing in an oven at 80 °C for 4 h. It is worth noting that liquid metals have an oxide layer that contributes to their extremely low vapor pressure, preventing volatilization, and they exhibit good biocompatibility.

Figure 2a depicts the fabrication process of the protrusion-structured LME, which is created using a templating method. The copper template with a negative mold is fabricated using a laser engraving machine, which was reported in our previous work. Briefly, chemical reagents are used to clean the copper template. Then, the LME solution is spin-coated on the copper template, and after solidification, it is peeled off the copper template to obtain a protrusion-structured LME. Figure 2b demonstrates the fabrication process of the supporting layer. PDMS solution was poured into the mold, and after curing at 80 °C for 4 h, the supporting layer is obtained. Figure 2c illustrates the fabrication process of the thermal collection layer. Employing a similar process as the supporting layer, a PDMS film with holes of the same size as the thermoelectric modules was prepared using 3D printing. The LME solution was poured into these holes and solidified to obtain the thermal collection layer.

The combination of various modules of FTD determines the overall stability and power generation efficiency. To ensure a stable bonding between the thermoelectric module and the LME, a semi-cured LME is used as an adhesive [19]. The LME solution is pre-cured at 60 °C for 10 min to significantly increase its viscosity. This viscous LME solution is then brushed to the interface between the thermoelectric module and the LME. Finally, curing at 80 °C for 4 h achieves the bonding between the thermoelectric module and the LME. The bonding between the supporting layer and the thermal collection layer is achieved using semi-cured PDMS solution. Specifically, the PDMS solution is pre-cured at 60 °C for 20 min to increase its viscosity. Subsequently, the viscous PDMS adhesive is applied to the interface between the supporting layer and the thermal collection layer, and curing at 80 °C for 4 h assembles the FTD. In addition, the use of rigid thermoelectric modules in the FTD may lead to potential reliability and durability issues. However, these problems can be mitigated through appropriate device selection and structural design. First, the shape of commercial rigid thermoelectric modules can be customized. By replacing square modules with elongated ones, the degree of conformity between the FTD and the targeted surface can be significantly increased. Secondly, there is a soft thermal collecting layer between the thermoelectric module and the target. This soft layer can be compressed to reduce delamination and stress concentration between the rigid thermoelectric module and the soft silicone, ensuring the stability of the structure and a tight attachment to the target.

**Figure 3:**
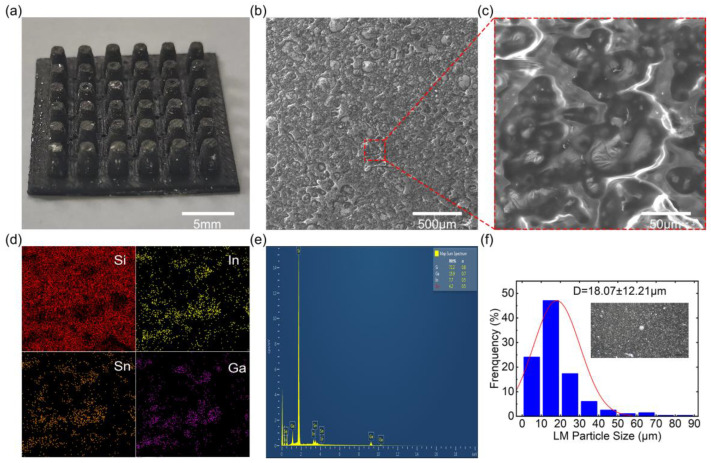
Characterization of LME. (**a**) Photo of LME; (**b**,**c**) SEM images of LME; (**d**,**e**) EDS characterization results of LME; (**f**) particle size analysis of LME.

The LME not only possesses flexibility but also exhibits a higher thermal conductivity, owing to the fluidity and metallicity of the LM [20]. Figure 3a presents the optical photograph of the protrusion-structured LME. The protrusion-structured LME appears black, with a visible presence of a small amount of LM particles on the surface, indicating the mixing of the LM within the PDMS. The morphology of the LME can be characterized using a scanning electron microscope (SEM), demonstrating irregularly distributed LM particles within the PDMS, as depicted in Figure 3b,c. Figure 3d presents the energy-dispersive X-ray spectroscopy (EDS) mapping, indicating the embedding of the LM particles within the PDMS. EDS analysis of the LME reveals the presence of gallium, indium, and silicon, consistent with the materials utilized in the preparation process, as shown in Figure 3e. Furthermore, Figure 3f displays the size distribution of the LM particles within the LME, where the diameters of the LM particles are concentrated within the range of 10 μm to 20 μm [21]. The LM can be cut into micron-sized particles through shear forces, which ensures the dispersion of the LM particles within the PDMS.

**Figure 4:**
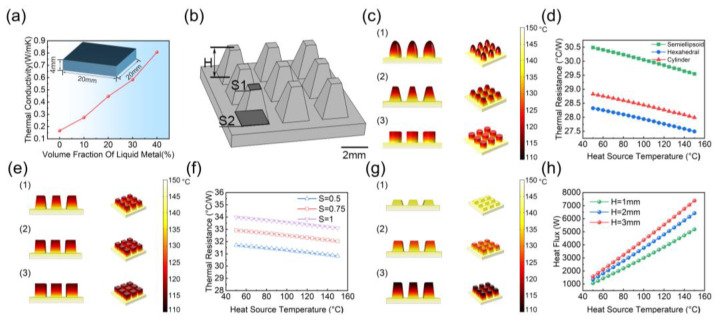
Simulation results of the LME structure on heat transfer performance. (**a**) Thermal conductivity test results of LME; (**b**) schematic diagram of the simulation model; (**c**) effect of LME protrusion shape (1: Semiellipsoid, 2: Hexahedral, 3: Cylinder) on temperature distribution; (**d**) effect of LME protrusion structure on thermal resistance; (**e**) effect of LME protrusion curvature (1: S = 0.5, 2: S = 0.75, 3: S = 0.5) on temperature distribution; (**f**) effect of LME protrusion curvature on thermal resistance; (**g**) effect of LME protrusion height on temperature distribution; (**h**) effect of LME protrusion height (1: H = 1 mm, 2: H = 2 mm, 3: H = 3 mm) on thermal resistance.

The LME possesses both flexibility and high thermal conductivity, making it an ideal heat-conducting material for FTD. Figure 4a illustrates the impact of different LM vol% on thermal conductivity. As the vol% of LM increases, the thermal conductivity continuously rises. At a 40 vol%, the thermal conductivity reaches 0.81 W/mK, representing a significant increase compared to pure PDMS by a factor of 0.16 W/mK.

The protrusion-structured LME increases thermal conduction between the thermoelectric modules and the air, which reduces the temperature in the cold end and elevates the open-circuit voltage of the thermoelectric modules [22]. To investigate the effectiveness of the protrusion-structured LME, we conducted a simulation analysis, as illustrated in Figure 4. A solid and fluid heat transfer field was selected for the simulation. As temperature changes occur gradually, we set the simulation type as steady state. An array of protrusion structures was established on a heating plate which was on the bottom and set as the heat source. The protrusion structures and the heating plate structure were designated as solid, while the rest was considered fluid. A heat source was applied on the heating plate with a heat flux which has a power of 1 W. Surface-to-environment thermal radiation of the protrusion structure array had an environmental temperature of 20 °C and a surface emissivity of 0.8. The geometric model of the protrusion structure array and the simulation setup are depicted in Figure 4b.

To investigate the design of the protrusion-structured LME on the heat dissipation temperature variation, we performed simulations to compare three structures: pyramid, cylinder, and semi-ellipsoid, as depicted in Figure 4c. With the same surface area in contact with the air domain, the temperature variations for the semi-ellipsoid, hexahedral, and cylinder are 33.4 °C, 31.7 °C, and 30.2 °C, respectively. The semi-ellipsoid structure exhibits the largest temperature variations in heat dissipation, which is attributed to the relatively larger air conduction heat transfer areas at the bottom of the semi-ellipsoid structure. Figure 4d shows the thermal resistance of LME with protrusions of different structures under different heat source temperatures. It can be seen that the hexahedron has the smallest thermal resistance, indicating that it has less obstruction to heat flow, and heat can pass through more easily and quickly, allowing heat to be effectively conducted from the heat source to the cooling medium or environment, thereby improving the power generation efficiency of the FTD. Figure 4e compares the temperature distribution of hexahedrons with different curvatures. The hexahedron LME with the maximum curvature has the smallest thermal resistance and the greatest gain in power generation efficiency for the FTD, as shown in Figure 4f. Figure 4g compares the impact of the height of hexahedron LME on temperature distribution. It can be seen that the tallest hexahedron LME has the largest surface temperature difference, meaning better heat dissipation performance. Figure 4h compares the impact of the height of hexahedron LME on heat flow under different heat source temperatures. At the same heat source temperature, the taller hexahedron LME has a higher heat flow, meaning a faster rate of heat diffusion, allowing heat to spread rapidly within the material. This is because the tallest hexahedron LME has a larger surface area in contact with the environment, which improves heat dissipation efficiency and results in an increase in heat flow.

**Figure 5:**
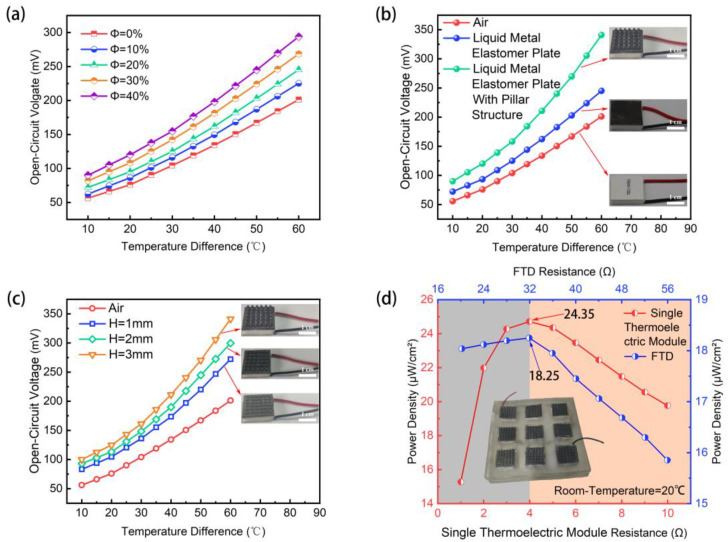
Performance testing of FTD. (**a**) The effect of liquid metal volume fraction on open-circuit voltage under different temperature differences; (**b**) the effect of different LME protrusion structures on open-circuit voltage under different temperature differences; (**c**) the effect of LME protrusion height on open-circuit voltage under different temperature differences; (**d**) the relationship between the internal resistance and power density of FTD and single thermoelectric module.

In Figure 5a, the effect of different vol% of LM on temperature difference versus open-circuit voltage is investigated. The open-circuit voltage of the thermoelectric module increases with the rise in vol% of LM. As for pure PDMS, the open-circuit voltage of the thermoelectric module decreases due to the inherently poor thermal conductivity. However, increasing the LM vol% enhances the thermal conductivity of the LME, consequently improving the open-circuit voltage of the thermoelectric module. The LME with 40 vol% LM has the highest open-circuit voltage (280 mV). Excessive LM may induce internal defects in the LME, reducing mechanical strength. As a result, we select the LME with 40 vol% LM to fabricate the protrusion-structured LME.

Figure 5b demonstrates the enhancement of protrusion structure on the performance of thermoelectric modules. For a temperature difference of 60 °C, the open-circuit voltage of the thermoelectric module is 190 mV with a pure elastomer. When an LME film is attached to the cold surface of the thermoelectric module, the open-circuit voltage at a 60 °C temperature difference increases to 240 mV. By encapsulating the thermoelectric module with the protrusion-structured LME, the open-circuit voltage at a 60 °C temperature difference significantly rises to 340 mV. This increase is attributed to the fact that the protrusion-structured LME substantially decreases the cold-end temperature of the thermoelectric module, which increases the temperature difference and the open-circuit voltage [6].

In Figure 5c, the influence of different heights of protrusion-structured LME on the open-circuit voltage is also investigated. Consistent with simulation results, as the height of the protrusion structure increases, the open-circuit voltage of the thermoelectric module steadily rises, reaching a maximum of 340 mV. This output is 1.7 times higher than that of a pure thermoelectric module and 1.4 times greater than that of a thermoelectric module covered with an LME film.

Figure 5d shows the relationship between power density and internal resistance for both the FTD and a single thermoelectric module. On a heating plate at 60 °C, the power density of both the FTD and the single thermoelectric module increases with internal resistance initially, then decreases. The lower *x*-axis and the left *y*-axis represent the resistance and power density of the FTD, respectively, while the upper *x*-axis and the right *y*-axis represent the resistance and power density of a single thermoelectric module. The maximum internal resistance of the FTD is 32 Ω, while that of a single thermoelectric module is 4 Ω. This is because the FTD consists of nine thermoelectric modules connected in series, and its internal resistance is the sum of the resistances of these modules. The maximum power density of the FTD is 18.25 μW/cm^2^, whereas the maximum power density of a single thermoelectric module is 24.35 μW/cm^2^. There may be three reasons for this discrepancy: First, when multiple thermoelectric modules are connected in series, the total resistance is the sum of each module’s resistance, which increases the overall internal resistance. Under the same voltage, the current will decrease, thereby reducing the power output. Second, the connections between the thermoelectric modules might introduce additional contact resistance, which does not generate power but leads to energy loss. Third, different thermoelectric modules may experience different temperature gradients, causing some to operate under non-optimal conditions, leading to a decrease in overall power density. This indicates that the maximum power aligns with the resistance value when the internal resistance matches the external load, as previously indicated by reference [10].

**Figure 6:**
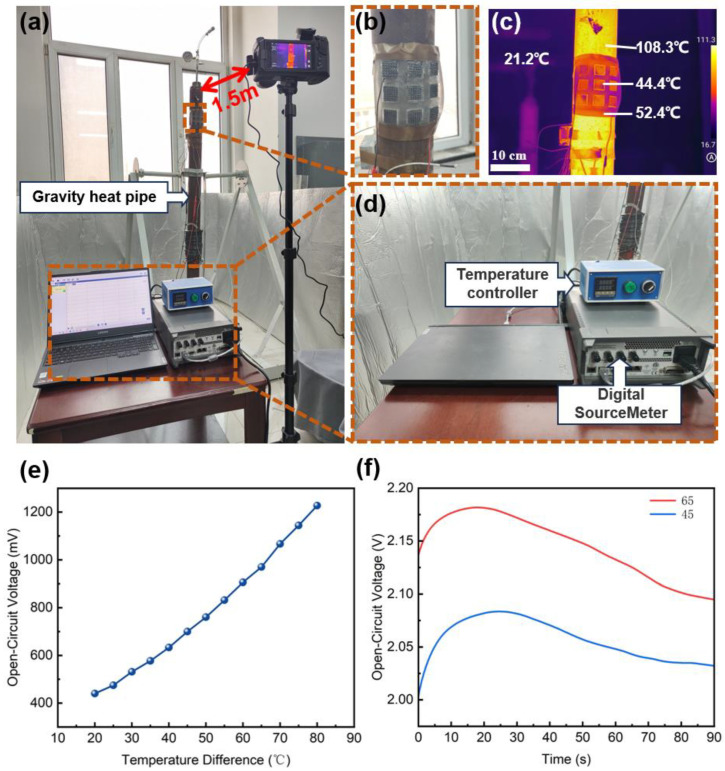
Experimental application of FTD on gravity heat pipe. (**a**) Experimental environment diagram; (**b**) fixed structure diagram of FTD and gravity heat pipe; (**c**) surface temperature distribution diagram of FTD and gravity heat pipe under heating state; (**d**) temperature control and experimental data acquisition equipment for gravity heat pipe; (**e**) relationship between FTD open-circuit voltage and temperature difference; (**f**) trend of FTD open-circuit voltage over time at the same temperature.

To demonstrate the feasibility of applying FTD to gravity heat pipes, we set up a gravity heat pipe experimental environment, as shown in Figure 6a. We used a 1.5 m high gravity heat pipe with a liquid composition of 95% water and 5% ethanol. Below the gravity heat pipe, a heating device connected to a controller was placed to simulate the spontaneous combustion of coal gangue. An infrared thermal imager was placed 1.5 m away from the FTD to record the temperature distribution. Figure 6b shows the assembly diagram of the FTD and the gravity heat pipe. The FTD was fixed to the surface of the gravity heat pipe with copper tape, with direct contact between the FTD and the gravity heat pipe. It can be seen that the FTD has good flexibility and can conform to the gravity heat pipe. The voltage data output by the FTD were collected through a digital multimeter connected to the computer, as shown in Figure 6d. The heat generated by the heating device causes the liquid in the gravity heat pipe to undergo a phase change, rapidly conducting the heat from the bottom to the top of the heat pipe, thereby cooling the coal gangue hill. Figure 6c shows the temperature distribution of the FTD and the gravity heat pipe after heating. It can be seen that the ambient temperature is 21.2 °C, the surface temperature of the gravity heat pipe can reach 108 °C, and the surface temperature of the FTD shows two temperatures of 44.4 °C and 53.4 °C. This is because the insulating layer of the FTD gathers heat to the position of the thermoelectric generating piece covered with high-thermal-conductivity LME, which reduces the temperature of the FTD surface, increases the temperature difference between the cold and hot ends of the FTD, and enhances the thermoelectric generating performance of the FTD [23]. Figure 6e shows the relationship between the open-circuit voltage of the FTD and the temperature difference. As the temperature difference between the FTD and the gravity heat pipe increases, the open-circuit voltage of the FTD shows a monotonic increasing trend. This is because a higher temperature difference leads to more carrier diffusion, thereby generating a higher electromotive force [24,25]. Figure 6f shows the trend of the open-circuit voltage of the FTD over time when the temperature difference is 65 °C and 45 °C. It can be seen that with the increase in time, the output voltage of the FTD first rises and then gradually decreases. This is because the FTD gradually reaches thermal equilibrium, that is, the temperatures of the cold and hot ends of the FTD become equal, and the heat transfer rate depends on the material, size, and temperature difference of the object.

**Figure 7:**
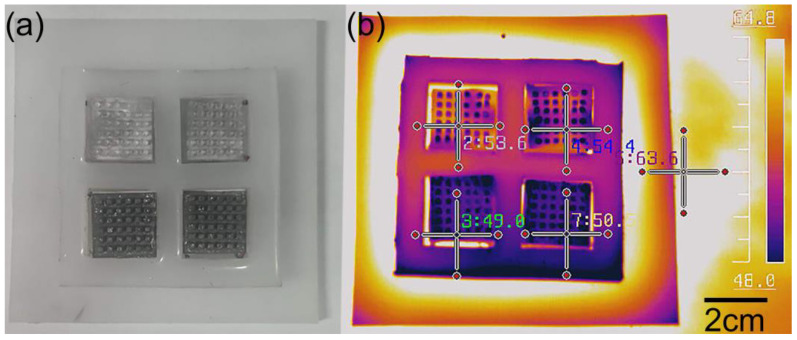
Demonstration of the temperature differential enhancement by the protrusion-structured LME. (**a**) Photograph of the FTD with and without the protrusion-structured LME. (**b**) Surface temperature distribution diagram of the FTD.

To demonstrate the enhancement of thermoelectric efficiency in the FTD by the protrusion-structured LME, we compared the temperatures of FTD samples with and without protrusion-structured LME coverage on a hot plate at 80 °C, as shown in Figure 7a. Infrared photos indicate that the temperature of the section covered with protrusion-structured LME was 49 °C, while the temperature of the section without protrusion-structured LME coverage was 63 °C, as illustrated in Figure 7b. Therefore, protrusion-structured LME can significantly reduce the temperature difference across the thermoelectric module, thereby improving the thermoelectric generation efficiency.

## 3. Conclusions

This work introduces an FTD with a protrusion-structured LME. We fabricated a thermal collection layer using a high-thermal-conductivity LME combined with an elastomer possessing lower thermal conductivity. Additionally, we analyzed the influence of LM vol% on the thermal conductivity of LMEs. To enhance the temperature difference between the hot and cold sides of the thermoelectric modules, we applied an LME with a protrusion structure. Employing finite element simulation, we examined the impact of the shape, spacing, and height of protrusion-structured LMEs on the temperature difference between the hot and cold sides of the thermoelectric modules. We manufactured a FTD with a protrusion-structured LME and compared the open-circuit voltages according to different LM vol%, shapes, and heights of the protrusion-structured LMEs. Moreover, we validated the potential application of the LME with a protrusion structure on a GHP.

## 4. Experimental Section

Materials: The gallium–indium–tin alloy (Galinstan) was procured from Shenyang Baijujie Scientific Instrument Co., Ltd. (Shenyang, China). PDMS (Sylgard 184) was sourced from Dow Corning (Midland, TX, USA). Various chemical agents, such as acetone, hydrochloric acid, and anhydrous ethanol, were obtained from Shanxi Youlaibo Chemical Technology Co., Ltd. (Shanxi, China). The thermoelectric modules were purchased from Shenzhen Taier Electronics Technology Co., Ltd. (Shenzhen, China).

Fabrication process: The PDMS base solution and curing agent were combined in a 10:1 ratio using a mixer to produce the pre-polymer for the PDMS. Varying vol% of LM were then mixed with the PDMS solution using a vacuum mixer at 2000 rpm for 2 min to prepare the LME suspension. Resin templates for the supporting layer and thermal collection layer were produced via three-dimensional printing. PDMS and LME solutions were individually poured into the resin templates to fabricate the supporting layer and thermal collection layer. The LME suspension was spin-coated onto the copper template at 370 rpm using a homogenizer, resulting in a smooth film with a thickness of 500 μm. All elastomers (PDMS and LME) were cured at 80 °C for 5 h. After reaching room temperature, the elastomers were removed from the template. A semi-cured LME solution was employed to bond the thermoelectric modules with the protrusion-structured LME. Semi-cured PDMS was used to bond the supporting layer and thermal collection layer.

Characterization: The SEM images of the LMEF were acquired using a focused ion beam double-beam scanning electron microscope (TESCAN LYRA 3, Brno, Czech Republic). Optical images were captured using a Canon camera. The thermal conductivity of LME was determined using a thermal conductivity meter (HCDR-S, Nanjing Huicheng Instrument Co., Ltd., Nanjing, China). Temperature distribution was assessed using an infrared thermal imager (D600, SATIR Trade UK Co., Ltd., Drogheda, Ireland). The copper template was crafted using a laser engraving machine (Changchun New Industries Optoelectronics Technology Co., Ltd., Changchun, China). The elastomer solution was blended using a vacuum mixer (HMV600, Shenzhen Hasai Technology Co., Ltd., Shenzhen, China). The LME film was fabricated using a spin coater (KW-4A, Institute of Microelectronics of The Chinese Academy of Sciences, Beijing, China).

## Data Availability

The original contributions presented in the study are included in the article, further inquiries can be directed to the corresponding author.
